# Mouse islet‐derived stellate cells are similar to, but distinct from, mesenchymal stromal cells and influence the beta cell function

**DOI:** 10.1111/dme.15279

**Published:** 2024-01-07

**Authors:** Wei Xu, Yunting Zhou, Tianyuan Wang, Chengming Ni, Chunlei Wang, Rui Li, Xuekui Liu, Jun Liang, Tzu‐wen Hong, Bo Liu, Aileen J. F. King, Shanta J. Persaud, Zilin Sun, Peter M. Jones

**Affiliations:** ^1^ Department of Endocrinology, Xuzhou Central Hospital Xuzhou Institute of Medical Sciences, Xuzhou Medical University, Xuzhou Clinical School of Nanjing Medical University, Affiliated Hospital of Medical School of Southeast University Xuzhou China; ^2^ Diabetes & Obesity, School of Cardiovascular and Metabolic Medicine & Sciences King's College London London UK; ^3^ Department of Endocrinology Nanjing First Hospital, Nanjing Medical University Nanjing China; ^4^ Endocrinology Department Air Force Hospital of Eastern Theater Command Nanjing China; ^5^ Department of Endocrinology, Zhongda Hospital Institute of Diabetes, Medical School, Southeast University Nanjing China; ^6^ Department of Endocrinology Yancheng Clinical College of Xuzhou Medical University Yanchen China

**Keywords:** diabetes mellitus, islet stellate cells, islets of Langerhans, mesenchymal stromal/stem cells

## Abstract

**Aims:**

Evidence is accumulating of the therapeutic benefits of mesenchymal stromal cells (MSCs) in diabetes‐related conditions. We have identified a novel population of stromal cells within islets of Langerhans – islet stellate cells (ISCs) – which have a similar morphology to MSCs. In this study we characterize mouse ISCs and compare their morphology and function to MSCs to determine whether ISCs may also have therapeutic potential in diabetes.

**Methods:**

ISCs isolated from mouse islets were compared to mouse bone marrow MSCs by analysis of cell morphology; expression of cell‐surface markers and extracellular matrix (ECM) components; proliferation; apoptosis; paracrine activity; and differentiation into adipocytes, chondrocytes and osteocytes. We also assessed the effects of co‐culture with ISCs or MSCs on the insulin secretory capacity of islet beta cells.

**Results:**

Although morphological similar, ISCs were functionally distinct from MSCs. Thus, ISCs were less proliferative and more apoptotic; they had different expression levels of important paracrine factors; and they were less efficient at differentiation down multiple lineages. Co‐culture of mouse islets with ISCs enhanced glucose induced insulin secretion more effectively than co‐culture with MSCs.

**Conclusions:**

ISCs are a specific sub‐type of islet‐derived stromal cells that possess biological behaviors distinct from MSCs. The enhanced beneficial effects of ISCs on islet beta cell function suggests that they may offer a therapeutic target for enhancing beta cell functional survival in diabetes.

**What's new?**

- In this study we have isolated an endogenous population of stellate cells from mouse islets (Islet Stellate Cells; ISCs) and compared their morphological and functional phenotypes to those of bone marrow‐derived mesenchymal stromal cells (MSCs).
- Our results demonstrate that ISCs are morphologically similar to, but functionally distinct from, MSCs. Importantly, co‐culture of mouse islets with ISCs enhanced glucose‐induced insulin secretion more effectively than co‐culture with MSCs.
- Our study suggests that ISCs may offer a therapeutic target for enhancing beta cell functional survival in diabetes.

## 1 INTRODUCTION

Mesenchymal stromal/stem cells (MSCs) were first described as non‐haemopoietic cells isolated from bone marrow with the capacity of self‐renewal and multipotency, and subsequent studies have identified similar cell populations in most vascularized tissues. MSCs adhere to tissue culture plastic substrates, express CD73, CD90, and CD105 and have the ability to differentiate into adipocytes, chondrocytes and osteocytes in vitro.^1^

The potential therapeutic benefits of MSCs in the treatment of diabetes have been well documented in recent years. For example, transplantation of bone marrow‐derived MSCs increased plasma insulin levels and reduced hyperglycaemic in a mouse model of diabetes^2^; and a recent clinical trial reports beneficial outcomes from a single infusion of MSCs in newly diagnosed type‐1 diabetes.^3^

MSCs have direct effects on islet beta cell function through secreted paracrine factors, such as Annexin‐A1 (ANXA1) and Stromal Cell‐Derived Factor‐1 (SDF‐1), which improve insulin secretion and protect beta cells from inflammatory cytokine‐induced apoptosis.^4^ Other MSC secretory products, including Fibroblast Growth Factor (FGF), TGF‐β1, VCAM‐1 and VEGF, improve the function of islet grafts via their immunomodulatory effects,^5^ or their ability to influence beta cell differentiation and maturation.^6^ We have previously reported that co‐transplantation of MSCs with islets in a mouse model of diabetes improves graft outcomes by maintaining islet structure and organization and improving revascularization. Some of these effects can be recapitulated in an MSC‐free model by simply pre‐culturing islets with a cocktail of MSC‐derived secretory products offering a potential route to improving the outcomes of human islet transplantation as a therapy for type 1 diabetes.^7^

Pancreatic Stellate Cells (PSCs) were first identified in 1998,^8^ and have since been implicated in the development of pancreatitis, pancreatic fibrosis, pancreatic cancer, and in normal pancreas development.^9^, ^10^ PSCs express stromal cell markers and are multipotent.^11^ Previous studies have reported the existence of stromal cell populations within the endocrine pancreatic islets which were referred to as islet‐derived fibroblast‐like cells,^12^ islet‐derived precursors,^13^ or islet‐derived progenitor cells,^14^ but the precise identity and biological properties of these populations have not yet been studied in detail. We have previously characterized a distinct population of PSCs that can be expanded from isolated rodent and human islets and identified them as islet stellate cells (ISCs). ISCs expanded from within the islets rather than from the islet periphery, consistent with an endogenous islet cell population, suggesting that they represent a distinct sub‐population of PSCs,^15^, ^16^ and they are activated by the diabetic environment,^17^ consistent with a role in islet responses to the development of diabetes. There is accumulating evidence that ISCs influence beta cell function within the islet microenvironment. Thus, an islet regenerating protein (Reg‐1) or the Wnt family member 5A (Wnt5a) regulated the activation of ISCs via signalling pathways including the phosphorylation of Akt, Erk and Smad^18^; islet dysfunction caused by vitamin A deficiency was attributed to the induction of ISC activation via retinol‐binding protein^19^; and in vitro co‐culture of isolated islets with ISCs modified their insulin secretory responses.^20^, ^21^

MSCs are currently under intense investigation for their therapeutic potential in regenerative medicine. Given the similarities between MSCs and ISCs we here assess the similarities and differences between these stromal cell populations to address whether ISCs may offer a useful target for treatments of diabetes.

## 2 MATERIALS AND METHODS

### 2.1 Animals

Male C567Bl/6 mice aged 8–12 weeks were purchased from Charles River Laboratories (Charles River, Margate, UK). All animal procedures were approved by our Institution's Ethics Committee and carried out under licence, following the UK Home Office Animals (Scientific Procedures) Act 1986.

### 2.2 Isolation and culture of mouse ISCs and MSCs

Mouse islets were isolated from db/m and db/db mice (12 weeks old, 6 mice per group; blood glucose levels >13 mmol/L) by type IV collagenase (1 mg/mL; Sigma, C4‐22, USA) digestion of the exocrine pancreas followed by purification on Histopaque 1077 (Sigma, 10771, USA) density gradients. The isolated islets were maintained in culture at 37°C (95% air/5% CO_2_), with most islets attaching to the dish within 3 to 7 days. ‘Passage 0’ is defined as 10 to 14 days after the islets were placed in culture at a time when the cultures were nearly confluent with stellate cells. Beginning at passage 0, cells were harvested with trypsin and sub‐cultured (1:2) every 3–4 days. Cells were maintained in Dulbecco's modified Eagle's medium (DMEM)/Ham's F12 (1: 1 v/v) (Sigma, D0547, USA) supplemented with 1% (v/v) penicillin/streptomycin solution (Gibco BRL, Gaithersburg, MD, USA) and 10% (v/v) fetal calf serum (FCS; Sigma‐Aldrich).^18^ Bone marrow MSCs were isolated from mice as described previously,^9^ The cell pellet was resuspended in DMEM/Ham's F12 supplemented with 1% (v/v) penicillin/streptomycin solution and 10% (v/v) fetal calf serum. The characterization of ISC and MSC populations between passages 3 and 8 showed consistent functional phenotypes, so all cells used in this study were confined to this range of passages.

### 2.3 Assessment of cell proliferation

The proliferation of ISCs and MSCs was assessed using an EdU assay (YF555 Click‐iT EdU Imaging Kits, C10338, UE).^18^ ISCs and MSCs were plated into a 6‐well plate at concentrations 1.0× 10^5^ cells/well. After formaldehyde fixation, cells were rinsed once with TBS and then stained by incubating for 10–30 min with 100 mM Tris, 0.5–1 mM CuSO_4_, 1–100 μM fluorescent azide and 50–100 mM ascorbic acid. The staining mix was prepared fresh each time and was used for staining cells immediately after addition of ascorbate. After staining, the cells on coverslips were washed several times with TBS with 0.5% Triton X‐100. EdU‐stained cells were immunostained using standard protocols. Cells were counterstained with Hoechst, mounted in standard mounting media and imaged by fluorescence microscopy. Three independent experiments were performed with observations within experiments in triplicate.

### 2.4 Apoptosis assay

An annexin V‐fluorescein isothiocyanate (FITC)/propidium iodide (PI) apoptosis detection kit (YF488‐Annexin V and PI Apoptosis Kit, V13241, UE) was used to analyse ISC and MSC apoptosis as described previously.^18^ Cells were harvested through trypsinization and washed twice with cold PBS (0.15 mol/L, pH 7.2). Cells were centrifuged at 3000 r/min for 5 min, the supernatant was discarded, and the pellet was resuspended in 1 × binding buffer at a density of 1.0 × 10^5^–1.0 × l0^6^ cells per mL. 100 μL of the sample solution was transferred to a 5 mL culture tube and incubated with 5 μL of FITC‐conjugated annexin V (Pharmingen) and 5 μL of PI (Pharmingen) for 15 min at room temperature in the dark. Four hundred μL of 1 × binding buffer was added to each sample tube, and the samples were analysed by FACS using Cell Quest Research Software (Becton Dickinson). Three independent experiments were performed with triplicate observations within experiments.

### 2.5 Cell expression of paracrine mediators

Measurements of TGF‐β1 (Mouse TGF‐β1 ELISA kit, EMC107b, NeoBioscience), VCAM‐1 (Mouse VCAM‐1 ELISA Kit, ab201278, Abcam) and VEGF (Mouse VEGF ELISA kit, EMC103, NeoBioscience) were performed in cell supernatants to evaluate differences between ISCs and MSCs. TGF‐β1, VCAM‐1, VEGF levels in cell supernatants of 1 × 10^6^ ISCs or MSCs were determined using ELISA kit according to the manufacturers' instructions. All samples were assayed six times.

### 2.6 Immunofluorescence microscopy of ISCs and MSCs

Immunofluorescence microscopy was performed as described previously^18^ to evaluate differences among ISCs outgrown from islets in the expression of insulin and glucagon (1:200, ab210560, ab10988, Abcam). The expression of α‐smooth muscle actin (α‐SMA) (1:200, CL488‐14395, Proteintech), Collagen type I (Col‐I) (1:200, ab21286, Abcam), Collagen type III (Col‐III) (1:200, ab7778, Abcam), Collagen type IV (Col‐IV) (1:200, ab19808, Abcam), Fibronectin (FN) (1:200, ab2413, Abcam) and Laminin (1:200, 65505, Proteintech) was assessed and compared between ISCs and MSCs. All immunocytochemical analyses were performed in triplicate on separate preparations.

### 2.7 Quantitative real‐time polymerase chain reaction (qRT‐PCR)

Analysis of mRNA expression by qRT‐PCR analysis of extracts of ISCs and MSCs was performed as described previously,^18^ using at least six biological replicates.

### 2.8 Western blot analysis

ISCs and MSCs were harvested, washed with cold PBS and lysed with ice‐cold lysis buffer supplemented with protease inhibitors as described previously^18^ followed by immunoblotting analysis using the following primary antibodies: anti‐α‐SMA (1:1000, CL488‐14395, Proteintech), anti‐Col‐I (1:1000, ab21286, Abcam), anti‐Col‐III (1:1000, ab7778, Abcam), anti‐Col‐IV (1:1000, ab19808, Abcam), anti‐FN (1:1000, ab2413, Abcam), anti‐Laminin (1:1000, 65505, Proteintech), anti‐TGF‐β1 (1:1000, ab215715, Abcam) and anti‐GAPDH (1:5000, G8795, Sigma, USA). All Western blot analyses were performed in triplicate on separate preparations.

### 2.9 Analysis of cell‐surface markers

ISCs and MSCs (5 × 10^5^) at P3 were incubated with 1 mg of phycoerythrin‐conjugated or fluorescein‐isothiocyanate‐conjugated mouse anti‐rat monoclonal antibodies (R&D Systems, Minneapolis, MN) for 1 h at 48°C. After washing with PBS at 400 g for 5 min, the stained cells were re‐suspended in 500 mL of ice‐cold PBS (supplemented with 10% fetal bovine serum and 1% sodium azide) and subjected to FACS analysis (Becton Dickinson). Approximately 10^4^ events were counted for each sample. The percentage of cells with positive signal was calculated using the FACSCAN program (Becton Dickinson, San Jose, CA).^22^ ISCs and MSCs were labelled with antibodies against the cell surface markers CD11 antigen‐like family member B (CD11B) (PE anti‐mouse CD11b, 101208, BioLegend), CD29 (anti‐mouse CD29, 120208, BioLegend), CD31 (PE anti‐mouse CD31, 102408, BioLegend), CD44 (PE anti‐mouse CD44, 103008, BioLegend), CD45 (APC anti‐mouse CD45, 103112, BioLegend), CD73 (APC anti‐mouse CD73, 127210, BioLegend), CD90.2 (PE anti‐mouse CD90.2, 105308, BioLegend) and LY6A/E (Anti‐mouse LY6A/E, 122506, BioLegend) and sorted. All FACS analyses were performed in triplicate.

### 2.10 Differentiation of ISCs and MSCs into adipocytes, chondrocytes and osteocytes

ISCs or MSCs were cultured in differentiation induction media according to the manufacturer's protocols (No: 7531, 7541, 7551, ScienCell Research Laboratories). Differentiated phenotypes were assessed using oil red O staining for adipocytes, von Kossa staining for osteocytes and Alcian Blue staining for chondrocytes.

Adipogenic induction medium comprised Iscove's modified Dulbecco's medium (12440053, Gibco), 10% FBS, 10 mM Glutamax (35050061, Gibco), 1 μM dexamethasone (Solarbio, Beijing, China), 0.5 mM isobutyl methyl xanthine, 5 μg/mL insulin and 60 μM indomethacin. Control and induced ISCs or MSCs were stained with Oil Red O (Sigma‐Aldrich) to observe lipid droplet formation. All samples were assessed in triplicate.

Osteogenic induction medium comprised IMDM,10% FBS, 0.05 mM ascorbic acid, 10 mM Glutamax (Gibco, Carlsbad, CA, USA), 100 nM dexamethasone (Solarbio, Beijing, China) and 10 mM β‐glycerol phosphate. Control and induced ISCs or MSCs were stained with AgNO_3_ (7761888, Sigma) then counter‐stained with hematoxylin (517282, Sigma). All samples were assessed in triplicate.

Chondrogenic induction medium comprised IMDM, 10% FBS, 100 nM dexamethasone, 10 mM Glutamax, 50 μg/mL ascorbic acid and 10 ng/mL TGF‐β1. Control and induced ISCs or MSCs were stained with Alcian blue (ab150662, Abcam) to identify acid mucopolysaccharide synthesis. All samples were assessed in triplicate.

### 2.11 Coculture of islets with ISCs or MSCs

Cells were divided into quiescent ISCs, quiescent MSCs, active ISCs and active MSCs for 48 h unless otherwise specified. A total of 2 × 10^5^ cells were seeded in six‐well plates and cultured as described above for 24 h to form a confluent monolayer. For direct‐contact islet‐cell coculture, 100 mouse islets were seeded directly onto cell monolayers, and the culture medium was switched to DMEM (Sigma) supplemented with 10% (vol/vol) FBS and 1% (vol/vol) pen‐strep. The cocultures were incubated for 48 h at 37°C and in 5% CO_2_. Activation of MSCs and ISCs was achieved as described previously,^19^ with the medium being replaced with DMEM supplemented with interferon‐γ (IFN − γ) and tumour necrosis factor‐α (Proteintech; 20 ng/mL each), followed by an additional 8 h culture.

### 2.12 Islet secretory function in vitro

Static incubation of islets was performed to assess insulin secretion levels in vitro. Islets were preincubated for 1 h in a physiological salt solution containing 2 mM glucose. Groups of three islets were subsequently transferred into 1.5‐mL microcentrifuge tubes and incubated at 37°C in a buffer containing 2 mM CaCl_2_, 0.5 mg/mL BSA and 2 mM or 20 mM glucose. After 1 h, the incubation medium was retained and stored at −20°C until it was assayed for insulin content as described previously.^23^

### 2.13 Statistical analyses

R 4.2.1 software was performed to conduct the measurement data, and statistical analysis used the two independent sample *t*‐test or ANOVA. Two‐way repeated‐measurement ANOVA was used with the Bonferroni post hoc test to analyze repeated measurements in the same islet populations at different time points. A *p* < value of 0.05 was considered statistically significant. All data are expressed as mean ± SEM.

## 3 RESULTS

### 3.1 Characterization of ISCs from isolated mouse islets

**FIGURE 1:**
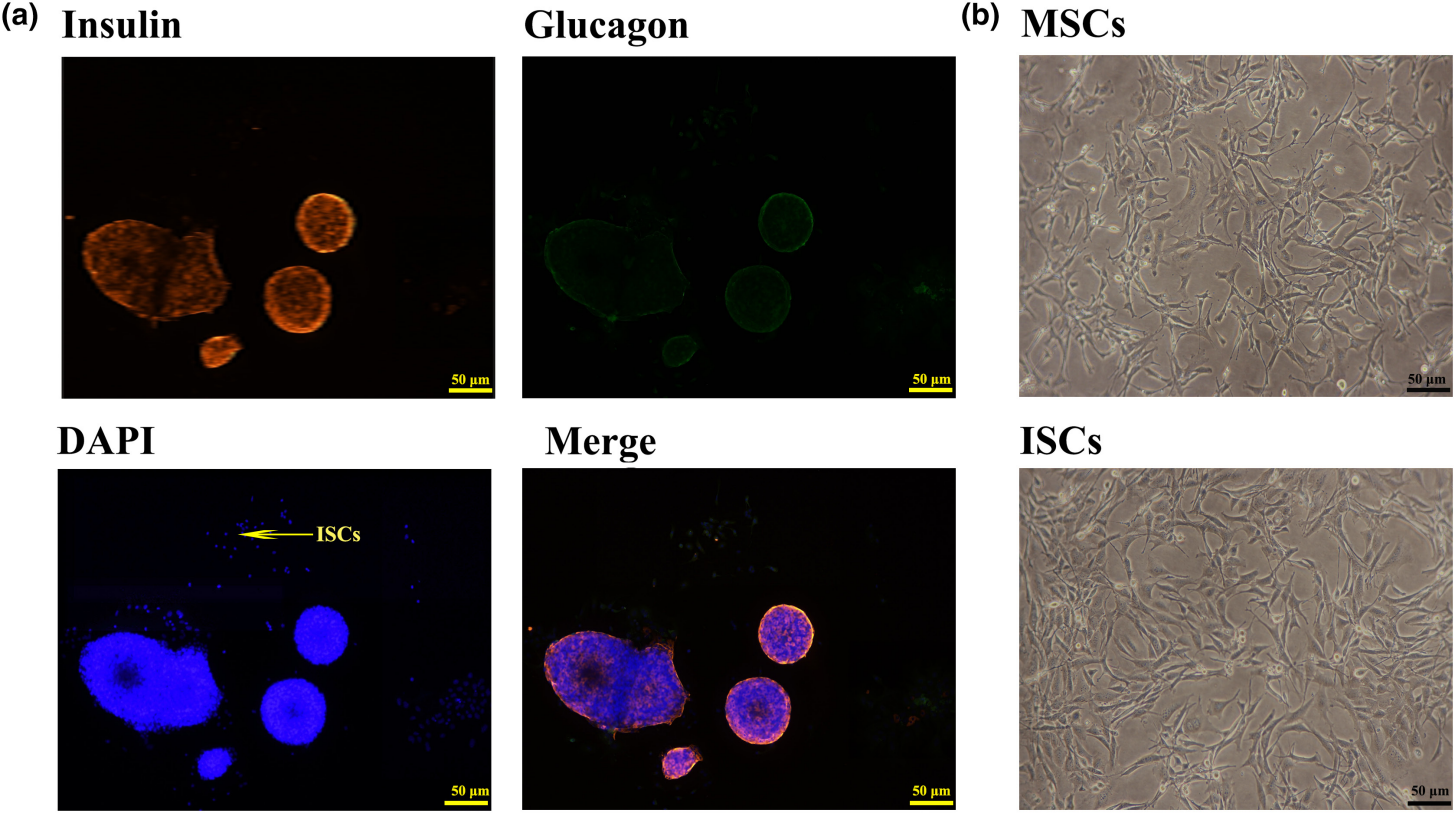
ISCs and MSCs have similar cell morphology. (a) Immunofluorescence staining of islets for insulin, glucagon and DAPI. There was no detectable expression of insulin or glucagon in the ISC outgrowth from islets; scale bar = 50 μm. (b) Light micrographs showed no obvious differences in the morphology of ISCs and MSCs; scale bar = 50 μm. All test analyses were performed in triplicate.

We performed immunostaining for insulin and glucagon in the cell outgrowth from islets to identify whether they originated from islet endocrine cells. As shown in Figure 1a the outgrowing cells were negative for both insulin and glucagon immunostaining suggesting that they were not insulin‐secreting β‐cells nor glucagon‐secreting α‐cells.

### 3.2 Cell morphology of ISCs and MSCs

We used light microscopy to compare the cell morphology of ISCs and MSCs (passage 3), during a 72‐h culture period after seeding 1.0 × 10^5^ cells/well in 6‐well microplates, as shown in Figure 1. After 12 h, “fibroblast‐like” ISCs adhered to the plastic tissue culture substrate but there were no obvious differences in overall morphology between the ISCs and MSCs (Figure 1b). The outgrowth of ISCs from islets increased over time during culture in vitro, as shown by the micrographs in Figure S1.

### 3.3 Differences in proliferation, apoptosis, secretion and the ECM component expression between ISCs and MSCs

**FIGURE 2:**
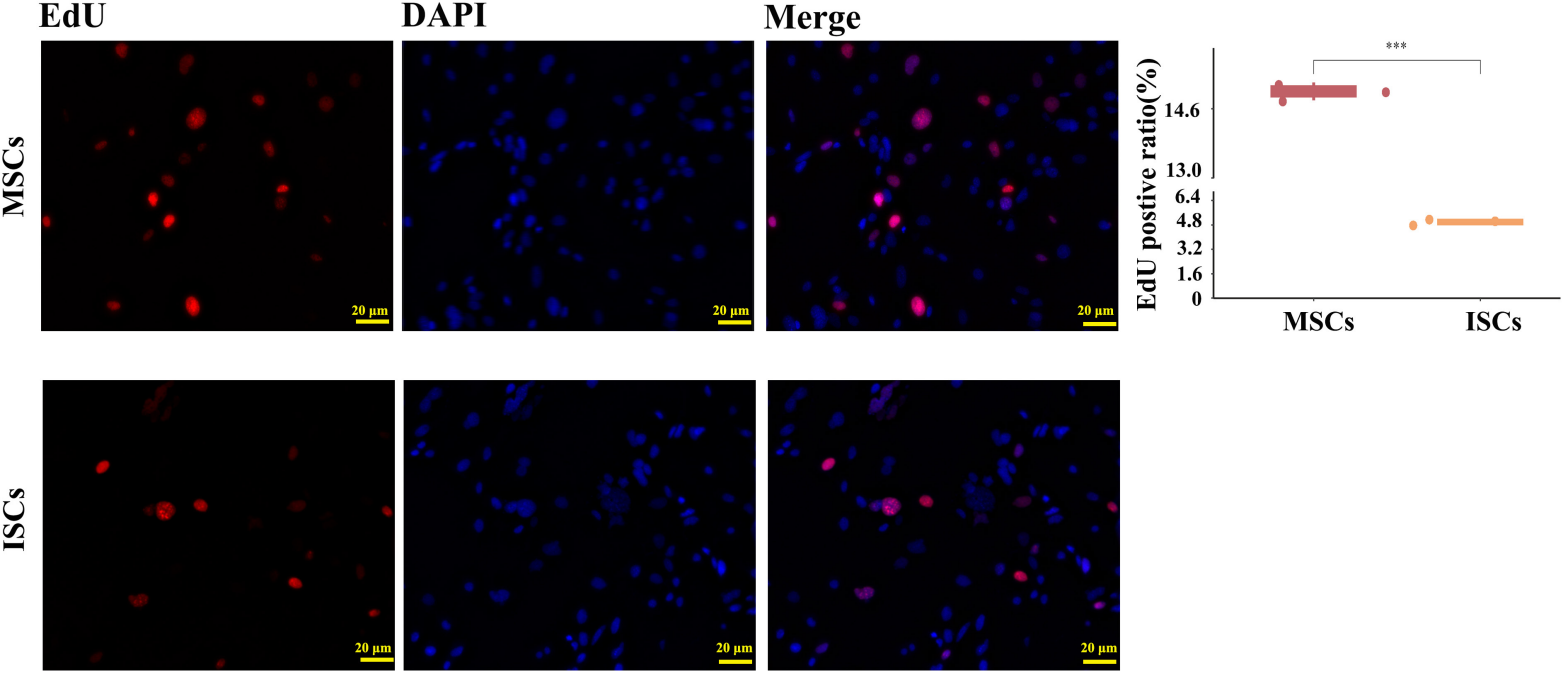
ISCs proliferate more slowly than MSCs. Immunostaining for EdU incorporation (red) showed less incorporation into ISCs Than into MSCs; scale bar = 50 μm. Quantification of EdU incorporation (right panel) showed a significantly higher incidence of proliferative cells in MSCs than in ISCs. ***p < 0.001. *p*‐Values were calculated using the Student's *t*‐test. All EdU assay analyses were performed in triplicate.

**FIGURE 3:**
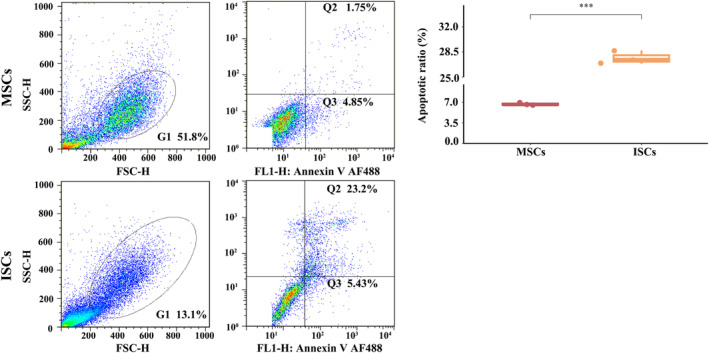
ISCs are more apoptotic than MSCs. Flow cytometric analysis based on annexin V‐fluorescein isothiocyanate (FITC)/propidium iodide (PI) detection of apoptosis showed more apoptotic cells in ISC populations than in than inequivalent MSC populations. Quantification of the proportion of apoptotic cells (right panel) showed a significantly higher incidence of apoptotic cells in ISCs than in MSCs. Data are expressed as the mean ± SE (*n* = 3); ***p < 0.001. All FCS analyses were performed in triplicate.

**FIGURE 4:**
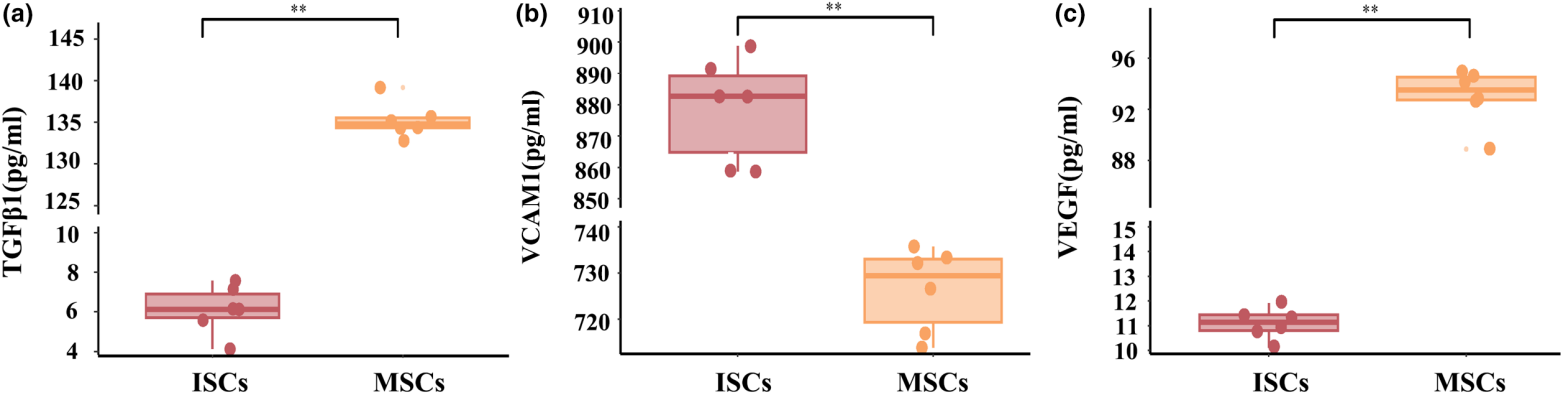
Differential secretory activities of ISCs and MSCs. Compared with MSCs, ISCs secreted significantly less TGF‐β1 (a) and VEGF (c), and significantly more VCAM‐1 (b). Data are expressed as the mean ± SE (*n* = 6); **p < 0.01 versus MSCs. The *p*‐values were calculated using the Student's *t*‐test. All measurements of secreted molecules were performed six times.

ISCs were less proliferative than MSCs in vitro, as shown in Figure 2, which shows much less EdU incorporation into ISCs (lower panels) when compared to similar cultures of bone marrow MSCs (upper panels). Flow cytometric analysis of apoptosis markers showed that ISCs were more apoptotic in vitro than equivalent MSC populations (Figure 3). Immunoassay measurements of paracrine secreted products (Figure 4) showed that ISCs secreted much less TGF‐β1 (Figure 4a) and VEGF (Figure 4c) than equivalent MSCs, and more VCAM‐1 (Figure 4b), consistent with different functional effects of the two cell types.

**FIGURE 5:**
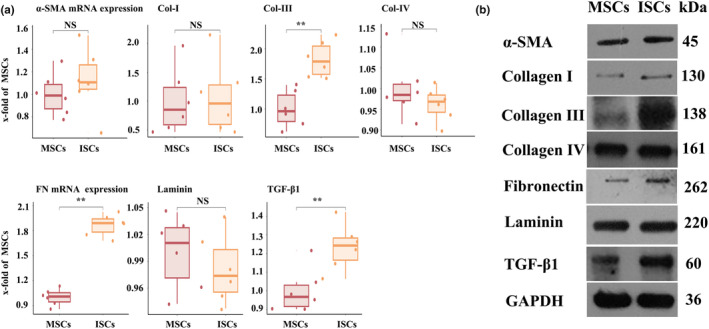
ECM component expression by ISCs and MSCs. (a) qRT‐PCR analyses of MSCs and ISCs were performed to measure mRNA levels of α‐SMA, Col‐I, Col‐III, Col‐IV, FN, Laminin and TGF‐β1. The expression of mRNAs for Col‐III, FN and TGF‐β1 was significantly higher in ISCs than in MSCs, with no statistically significant differences in the expression of mRNAs for α‐SMA, Col‐I, Col‐IV or Laminin between the two cell populations. Data are expressed as the mean ± SE (*n* = 6); **p < 0.01 vs. MSCs. The *p* values were calculated using the Student's *t*‐test. All RT‐PCR analyses were performed six times. (b) α‐SMA, Col‐I, Col‐III, Col‐IV, FN, laminin and TGF‐β protein levels were quantified in ISC and MSC extracts by immunoblotting. Protein levels of Col‐III, FN and TGF‐β1 were higher in ISCs than in MSCs, with no detectable differences in protein expression of α‐SMA, Col‐I, Col‐IV or Laminin between the two cell populations. All test analyses were performed in triplicate.

We examined the expression of ECM components in ISCs and MSCs by qRT‐PCR, immunoblotting and immunofluorescence. The mRNA and protein expression of α‐SMA, Col‐I, Col‐III, Col‐IV, FN, Laminin and TGF‐β1 in MSCs and ISCs was assessed by qRT‐PCR and immunoblotting, as shown in Figure 5. Both the mRNA and protein levels of Col‐III, FN and TGF‐β1 were higher in ISCs than in MSCs (Figure 5a,b, respectively), with no detectable differences in mRNA or protein expression of α‐SMA, Col‐I, Col‐IV or Laminin between the two cell populations. These observations were largely supported by immunofluorescence microscopy as shown in Figures S2 and S3, in which SMA and Col‐I immunoreactivities were higher in MSCs (Figure S2), while Col‐III (Figure S2), FN and Laminin (Figure S3) immunoreactivities were higher in ISCs than in MSCs. Together these observations suggest important phenotypical differences between ISCs and MSCs, consistent with a local intra‐islet role for ISCs in the maintenance of islet morphology and function.

### 3.4 Differences in cell‐surface markers and between ISCs and MSCs

**FIGURE 6:**
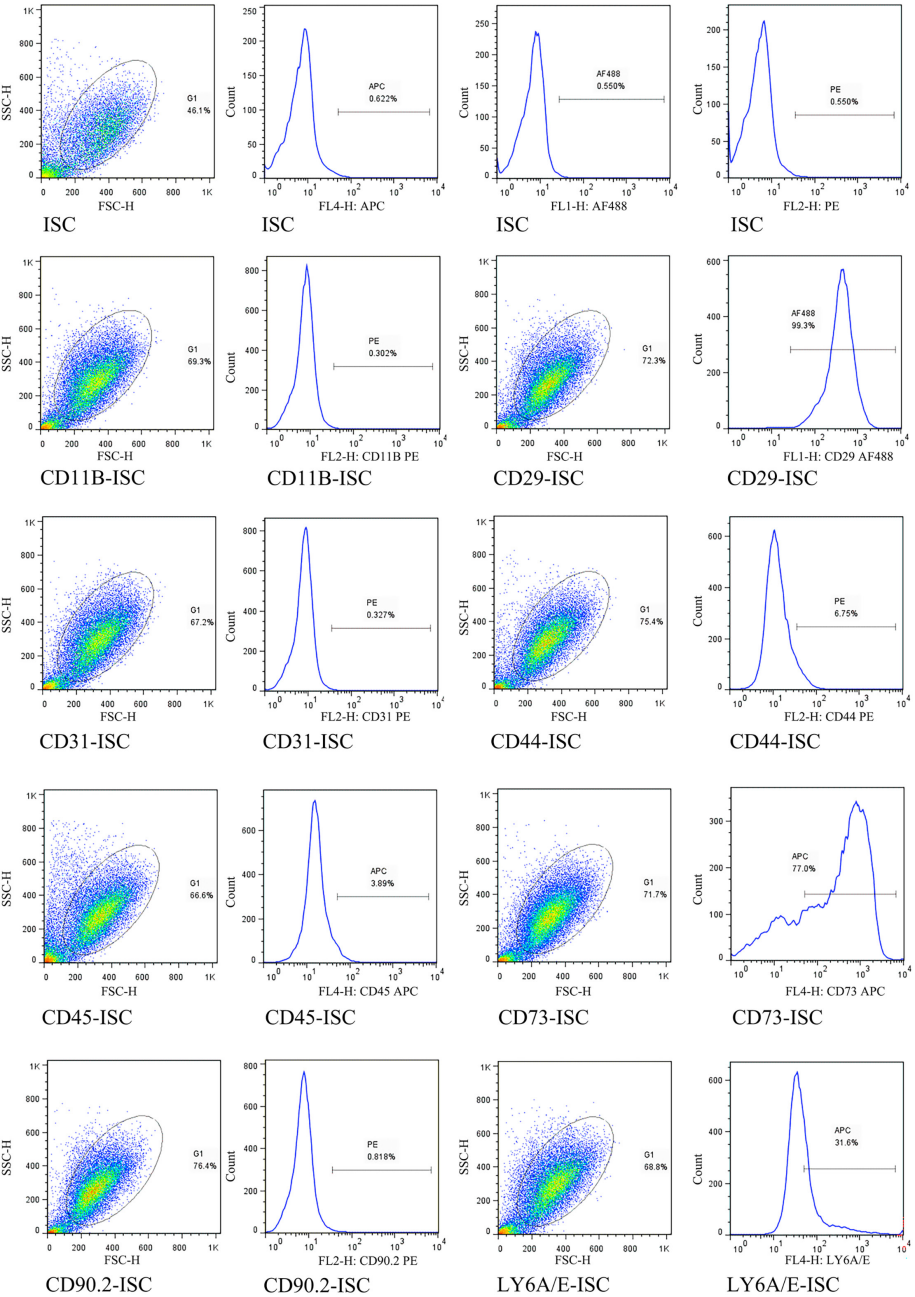
Analysis of ISC cell‐surface markers. Freshly isolated ISCs were stained with antibodies as indicated and analyzed by flow cytometry, demonstrating that ISCs have a CD11B‐, CD29+, CD31− and CD90− phenotypic profile. However, CD44 (6.75%), CD45 (3.89%), CD73 (77%) and LY6A/E (31.6%) were detected in ISCs but not in MSCs (Figure S3). APC, allophycocyanin; FITC, fluorescein isothiocyanate; PE, phycoerythrin. All test analyses were performed in triplicate.

We used FACS to evaluate differences in cell surface markers between ISCs (Figure 6) and MSCs (Figure S4). MSCs are conventionally identified by the surface marker phenotype CD11B−, CD29+, CD31−, CD44+, CD45−, CD73+, CD90− and LY6A/E+, as confirmed by our FACS analysis in Figure S3. Figure 6 shows FACs analysis of cell surface markers in ISCs demonstrating that our ISC populations have a CD11B−, CD29+, CD31−, and CD90− phenotypic profile. However, CD44 (6.75%), CD45 (3.89%), CD73 (77%), and LY6A/E (31.6%) were detected in ISCs but not in MSCs, suggesting that the cell surface marker expression in the ISC population differs from that of MSCs to some extent, confirming that ISCs and MSCs are phenotypically different cell populations and suggesting that ISCs are a islet‐specific stromal cell population.

### 3.5 Differentiation of ISCs into adipocytes, chondrocytes and osteocytes

**FIGURE 7:**
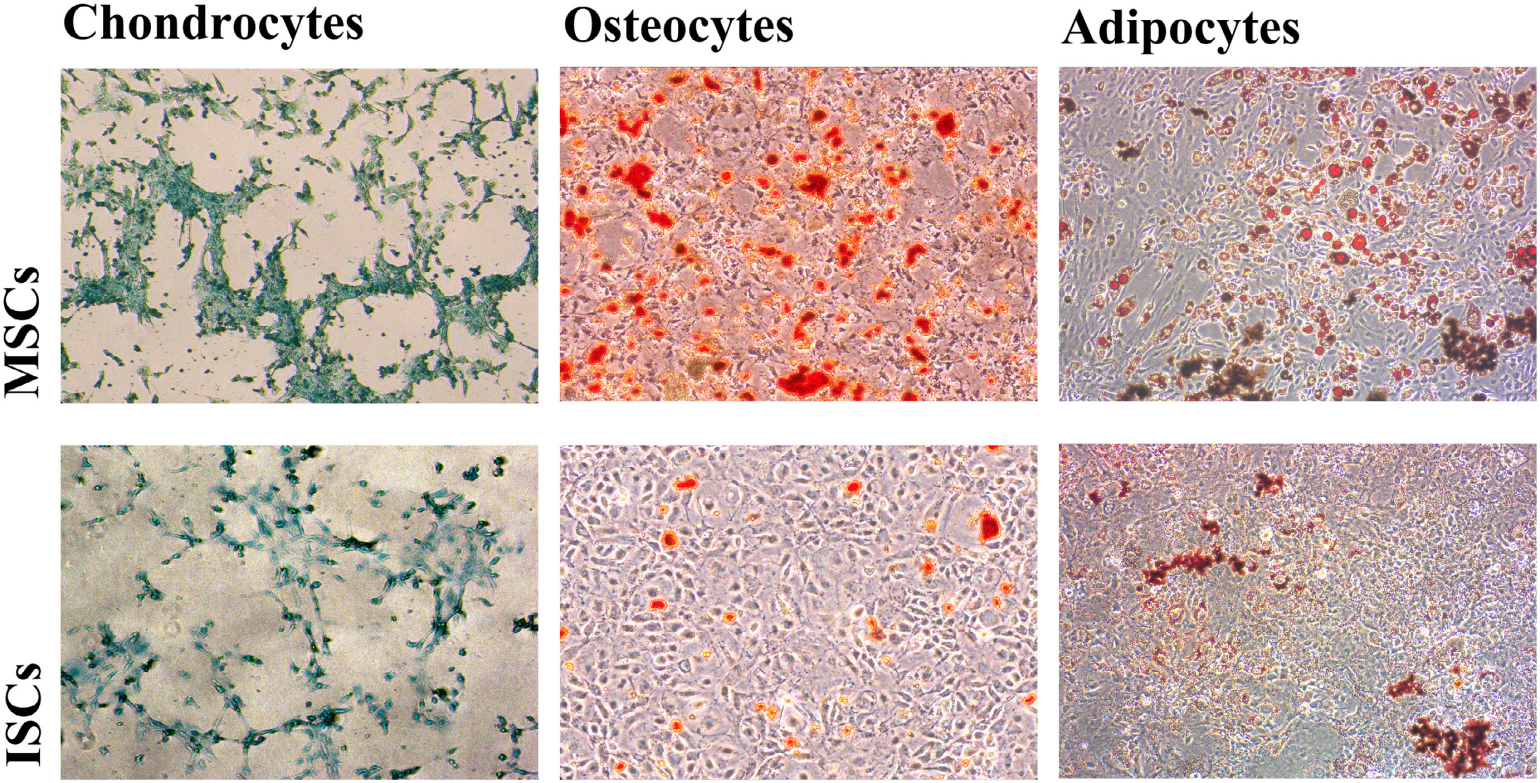
Differentiation of ISCs and MSCs into chondrocytes, osteocytes and adipocytes. MSCs (upper panels) and ISCs (lower panels) were capable of differentiating into chondrocytesosteocytes and adipocytes Photomicrographs showing Alcian Blue staining of MSCs and ISCs after differentiation into chondrocytes (a), von Kossa staining after differentiation into osteocytes (b) and oil red O staining after differentiation into adipocytes (c). The differentiation of ISCs into adipocytes, chondrocytes or osteocytes was generally less efficient than that of MSCs. All test analyses were performed in triplicate.

MSCs are defined as multipotent cells by their ability to differentiate into adipocytes, chondrocytes and osteocytes, so we investigated the ability of ISCs to differentiate down these lineages in vitro. Figure 7 shows that both ISCs (lower panels) and MSCs (upper panels) could be induced to express lineage specific characteristics, although this was less efficient in ISCs under the in vitro differentiation conditions applied in the current non‐quantitative study. This supports the notion that ISCs are an islet‐specific stromal cell population with characteristics distinct from the general MSC population.

### 3.6 Effects of ISCs and MSCs on the islet function

**FIGURE 8:**
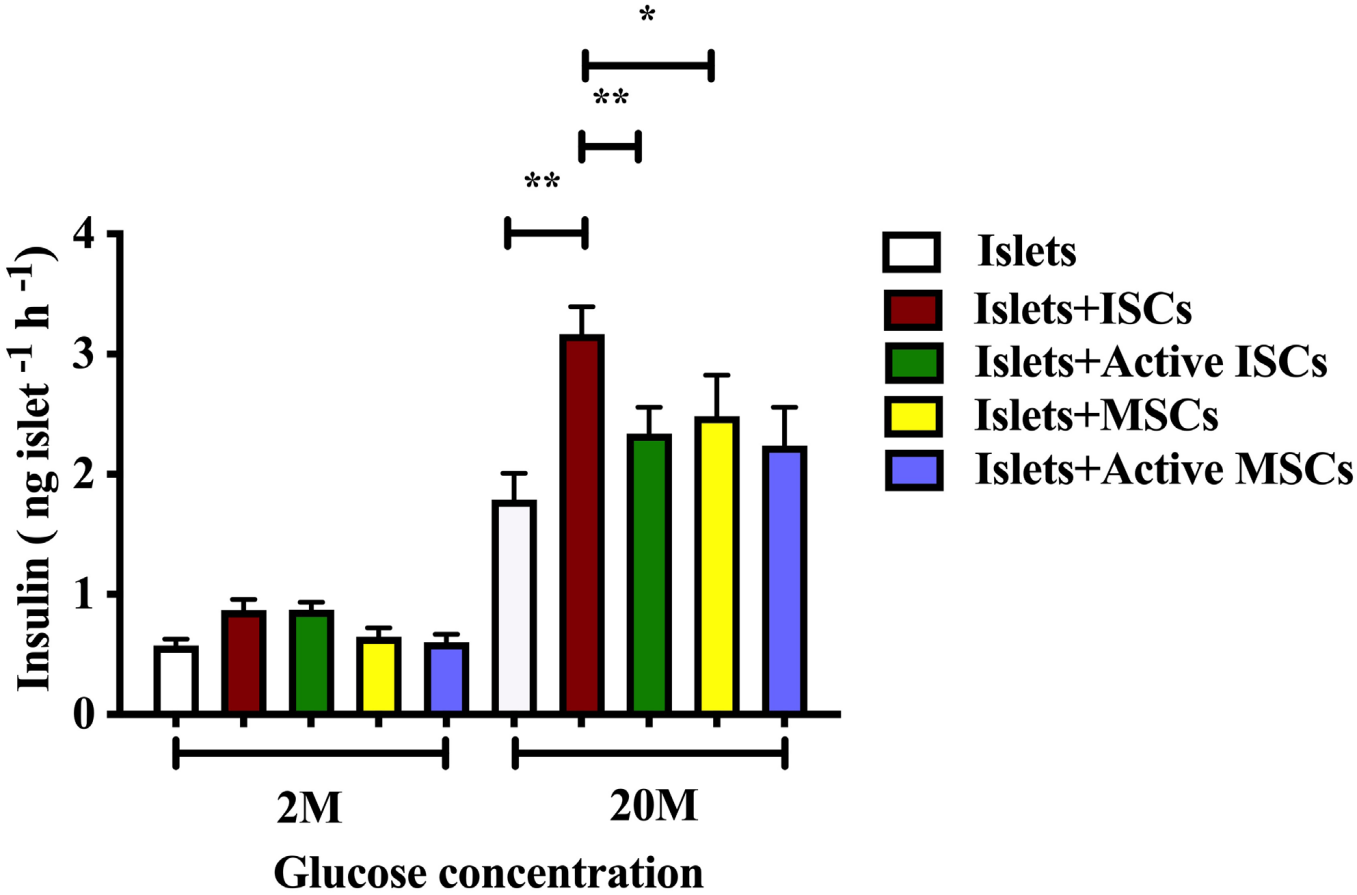
Effects of coculture with ISCs or MSCs on glucose‐induced insulin secretion from isolated mouse islets in vitro. Insulin release at 2 mM glucose and 20 mM glucose using 10 replicates of 3 islets per microcentrifuge tube: Islets were precultured alone with ISCs or active ISCs using a direct contact culture configuration; or with MSCs or active MSCs using a direct contact culture configuration. The activation of MSCs and ISCs was achieved by culture with interferon‐γ (IFN−γ) and tumour necrosis factor‐α (Proteintech; 20 ng/ml each), followed by an additional 8 h culture before use in experiments. *p < 0.05 and **p < 0.01 vs. islets cultured alone at the same glucose concentration. The *p* values were calculated using two‐way ANOVA with the Bonferroni post hoc test.

Co‐culture of isolated mouse islet with monolayers of mouse ISCs or MSCs enhanced glucose‐induced (20 mM) insulin secretion without affecting basal levels of insulin secretion (2 mM glucose) as shown in Figure 8. Glucose‐induced insulin secretion from islets co‐cultured with ISCs was significantly higher than from islets co‐cultured with MSCs and was further enhanced when ISCs were not pre‐activated by inflammatory cytokines. Activation had no effect on the ability of MSCs to enhance insulin secretion, as we have reported previously.^18^ These functional data demonstrate that endogenous ISCs located within islets may be involved in the regulation of insulin secretion.

## 4 DISCUSSION

Our original isolation of ISCs from rodent and human islets^15^, ^16^ and their subsequent characterization as a subpopulation of stellate/stromal cells with phenotypical traits distinct from those of pancreatic stellate cells^19^, ^20^, ^21^ raised the question of the possible function(s) of ISCs within the endocrine pancreatic islets. In the current study we have compared endogenous mouse islet ISCs with classical MSCs which have been reported to have beneficial influences on islet endocrine cell function in numerous experimental studies.^4^, ^7^, ^8^ We demonstrate that ISCs have morphological similarities to MSCs but have a distinct functional phenotype in terms of proliferation, apoptosis and the expression of ECM components and paracrine secretory products consistent with a localized function withing the microenvironment of the islet. In accordance with this, ISCs were more effective than bone marrow‐derived MSCs in enhancing glucose‐stimulated insulin secretion from isolated mouse islets, identifying ISCs as an endogenous islet stromal cell population with the capacity to regulate beta cell function. To our knowledge this is the first study to identify ISCs as potential therapeutic targets for the prevention or treatment of diabetes.

MSCs are a type of stromal cell named for their ability to differentiate into mesenchymal tissues and are fibroblast‐like cells capable of secreting growth factors and cytokines involved in haematopoietic and other processes.^24^ Their regenerative and anti‐inflammatory properties have been harnessed in the treatment of a range of disorders, including graft‐versus‐host disease, multiple myeloma, osteoarthritis and diabetes.^25^ In the field of diabetes‐related research, MSCs are known to promote the regeneration of pancreatic islet β cells, protect endogenous pancreatic islet β cells from apoptosis, and ameliorate insulin resistance in peripheral tissues by providing a supportive niche microenvironment driven by the secretion of paracrine factors or the deposition of ECM components.^26^, ^27^, ^28^, ^29^

Our comparison of ISCs with MSCs suggest that ISCs possess similar properties to MSCs and may therefore also have therapeutic uses in treating diabetes. Our previous studies demonstrated that ISCs regulate insulin secretion and apoptosis in the MIN6 insulinoma cell line via the secretion of Wnt5a and activation of the FoxO1‐PDX1‐GLUT2‐insulin signaling cascades.^20^, ^21^ We here confirm that ISCs isolated from mouse islets are more effective than bone marrow‐derived MSCs in enhancing glucose‐stimulated insulin secretion from primary mouse β‐cells in isolated islets. The underlying mechanisms of this effect of ISCs is uncertain but it is likely to involve the secretion of biologically active factors and the deposition of ECM, both of which have been implicated in the beneficial effects of MSCs on the islet function.^7^, ^23^

It is well documented that ECM plays important roles in the regulation of β‐cell function, the maintenance islet architecture and in the differentiation of stem cells into insulin‐secreting cells.^29^, ^30^, ^31^ Islet ECM is largely derived from vascular endothelial cells and perivascular cells,^32^ and collagen and laminin are key ECM components of the peripheral and internal basement membrane of the islets.^33^ We here demonstrate that ISCs express some ECM components such as α‐SMA, Col‐I, Col‐IV and Laminin at similar levels to MSCs, with higher expression levels of other components including Col‐III and FN. This is consistent with ISCs being involved in the regulation of β cell function through the secretion of ECM components within the islet microenvironment.

We demonstrated that, in common with MSCs, ISCs are multipotent with the ability to differentiate down adipocyte, chondrocyte and osteocyte lineages in vitro. There is some evidence that stromal cells such as MSCs also have the potential to differentiate towards an insulin‐producing cell phenotype with the expression of β cell‐associated transcription factors and functional elements such as pancreatic and duodenal homeobox 1 (Pdx1), paired box4 (Pax4), neurogenin 3 (Ngn3), and glucose transporter 2 (Glut2).^34^ For example, mouse islet‐derived fibroblast‐like cells were induced to express CD‐45 and PDX‐1 when treated with epidermal growth factor (EGF), platelet‐derived growth factor‐BB, and leukemia inhibitory factor.^14^ Similarly, stromal cells derived from isolated human islets expressed β‐cell genes when treated with fibroblast growth factor 2 (FGF2) and EGF.^35^ It was beyond the scope of the current study to address the directed differentiation of ISCs towards an endocrine phenotype, although our demonstration of their multipotency is consistent with this possibility.

In summary, ISCs are islet‐derived stromal cells which have some morphological and functional similarities with MSCs but have sufficiently different biological functions to be considered as a separate type of stromal cell. Their ability to enhance β‐cell secretory function in vitro and to synthesize and secrete ECM components identifies them as an important intra‐islet population with the potential to regulate the islet microenvironment. These characteristics suggest that ISCs may offer a potential therapeutic target for the treatment of diabetes.

## AUTHOR CONTRIBUTIONS

Writing—original draft preparation: WX and YT Z; writing—review and editing: PM J; project administration: PM J; All authors have read and agreed to the published version of the manuscript. PM J is responsible for the integrity of the work as a whole. WX, YT Z, TY W and CM N contributed equally to this work.

## CONFLICT OF INTEREST STATEMENT

The authors declare that they have no competing interests as defined by diabetic medicine or any other interests that might be perceived to influence the results and discussion reported in this paper.

## ETHICS STATEMENT

All animal procedures were approved by our institution's ethics committee and performed under license, in accordance with the U.K. Home Office Animals (Scientific Procedures) Act 1986.

## Supporting information


Figure S1.

Figure S2.

Figure S3.

Figure S4.

Data S1.
