# Supplementary material for: Mouse islet‐derived stellate cells are similar to, but distinct from, mesenchymal stromal cells and influence the beta cell function
Source: Diabet Med. 2024 Jan 7;41(6):e15279. doi: 10.1111/dme.15279 (PMC11451341; doi:10.1111/dme.15279)
Supplement: Supplementary file 1 — Figure S1. [file DME-41-e15279-s003.docx]

**
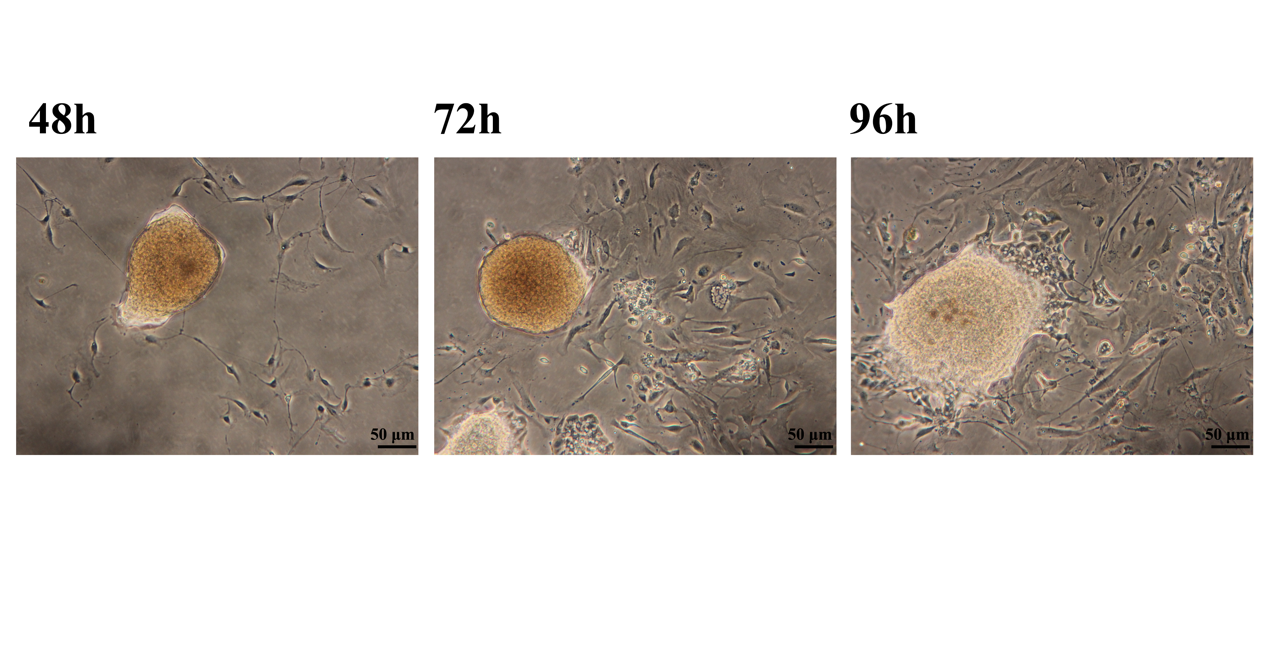
**

**Figure S1: ISC outgrowth from islets during culture in vitro.**

light microscopy data show the time-dependent outgrowth of ISCs from islets during in vitro culture. Scale bar = 50μm.
