# Supplementary material for: Mouse islet‐derived stellate cells are similar to, but distinct from, mesenchymal stromal cells and influence the beta cell function
Source: Diabet Med. 2024 Jan 7;41(6):e15279. doi: 10.1111/dme.15279 (PMC11451341; doi:10.1111/dme.15279)
Supplement: Supplementary file 2 — Figure S2. [file DME-41-e15279-s005.docx]

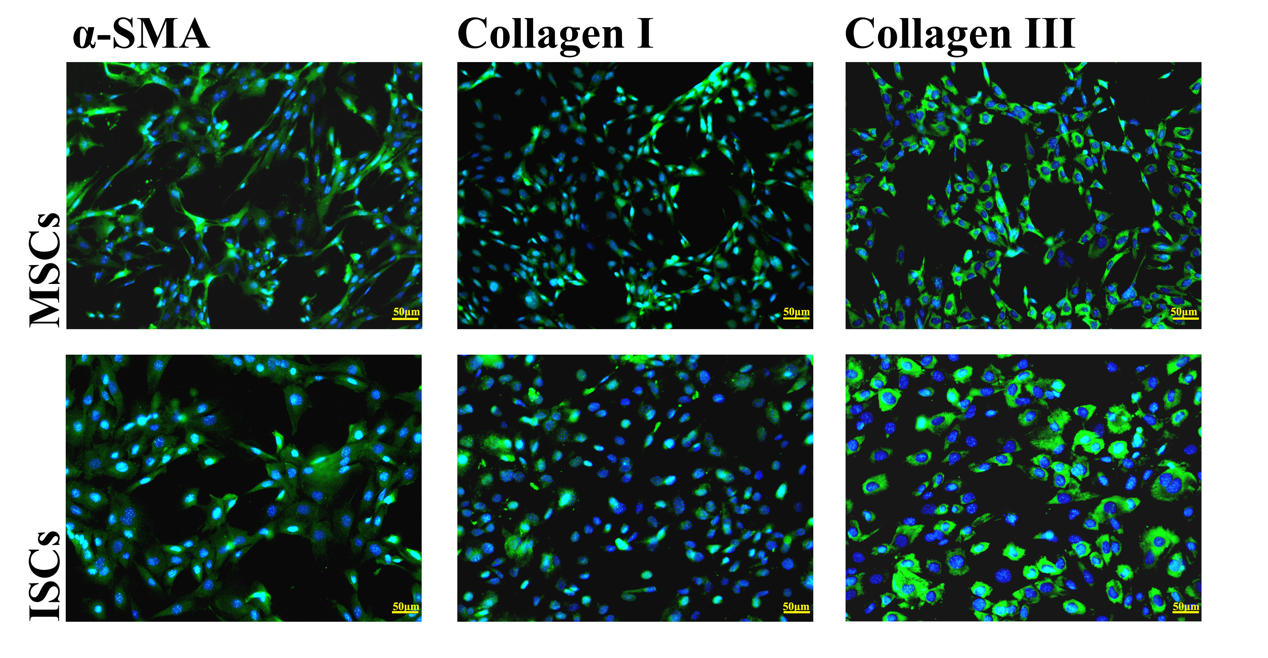


**Figure S2: Expression of ECM components by ISCs and MSCs.**

Immunofluorescence staining of MSCs (upper panels) and ISCs (lower panels) for α-SMA, Col-I and Col-III. Scale bar = 50 μm.
