# Supplementary material for: Mouse islet‐derived stellate cells are similar to, but distinct from, mesenchymal stromal cells and influence the beta cell function
Source: Diabet Med. 2024 Jan 7;41(6):e15279. doi: 10.1111/dme.15279 (PMC11451341; doi:10.1111/dme.15279)
Supplement: Supplementary file 4 — Figure S4. [file DME-41-e15279-s004.docx]

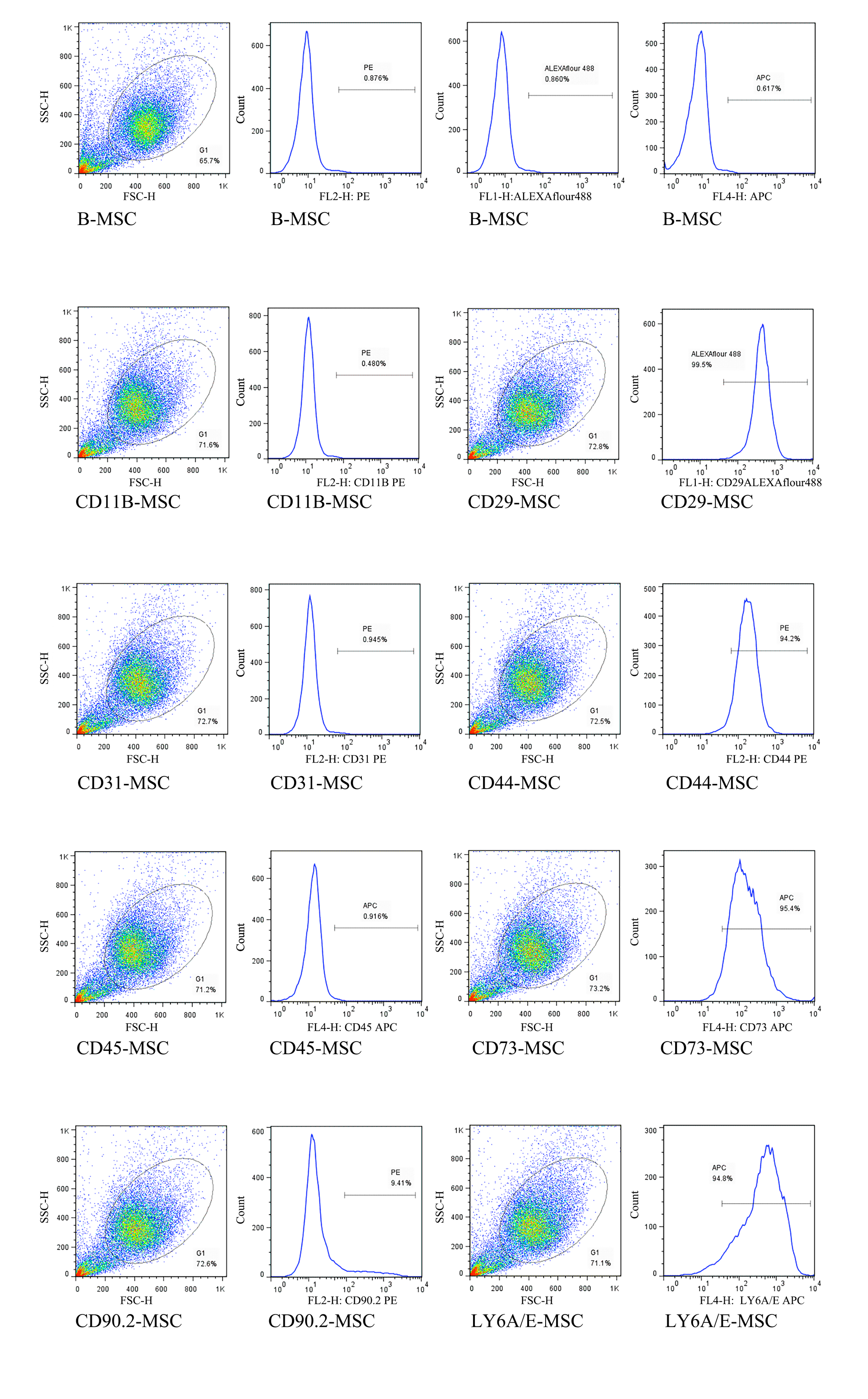


**Figure S4: Analysis of MSC cell‐surface markers.**

Fluorescence-activated cell sorting (FACS) analysis of MSCs for expression of CD11B, CD29, CD31, CD44, CD45, CD73, CD90.2, and LY6A/E. In each panel, cells treated with an antibody are indicated with a dotted line, and MSCs treated with CD marker‐specific antibodies are indicated with solid or dashed lines, respectively. Abbreviations: APC, allophycocyanin; FITC, fluorescein isothiocyanate; PE, phycoerythrin.
